# Method of Making Sets of Teeth with Continuous Gums

**Published:** 1853-10

**Authors:** C.F. Delabarre


*Method of making Sets of Teeth with Continuous Gums, as practiced by C. F. Delabarre*—This is taken from Delabarre's work, published in 1820. Translated from the French by Dr. F. Badarague, of Va., and corrected by Dr. E. Wildman, of Philadelphia.

We give merely that portion of the article which relates to the modus operandi and the ingredients used, as published in the April No. of the Dental News-Letter for 1853. Every thing of this kind is at this time interesting to the profession, and it is a matter of some moment to get the correct history of this method of inserting teeth from its first incipient stage to the present.

C. F. Delabarre remarks that, "The following composition embraces all the conditions required: Porcelain paste, 7 parts; calcined gypsum,* 1; white sand, (quartz,) 1-20th of the mass; such oxide as you desire, 150 gram. per kilo. Grind perfectly fine.

"Having obtained the result sought for, I make the following application of it; committing to the genius of the artist the variety of the means by which it may be executed.

"§ 2—*Mineral Veneering, or inlaid work.*—I make use of very thin teeth without cramps, (platinas) which have been baked at the heat of the porcelain furnace. I give them the proper form of teeth on a grind-stone; therefore this is a kind of inlaid work.

"When I wish to make a set of teeth with gums, I take the above-mentioned paste and place it upon the model, and then let it dry; after which I cut in the front of it little excavations, in which I fit the teeth, gluing them there with a little gum water; I set them with a small strip or roll of the paste, which I carve in imitation of the festoons of the gums; after which I cover it with a light coat of porcelain enamel,

* The more you add of gypsum, the more fusible the paste will be.

equally fusible by the addition of gypsum, and sometimes a little of the crystal of *Mont Cenis.*

"The teeth unite with the base so firmly that, after having been baked, the whole may be broken by a blow, but the parts already united will not be separated.

"It is well to secure the solidity of a set when it is of some considerable size, by attaching to it, before baking, a band of platina extending throughout its whole length, which would prevent the work from falling to pieces, should it crack in the furnace, and it would also facilitate its reunion by the application of enamel and a repeated baking in the furnace. Again, if one or more of the adjusted teeth should become deranged, it would be easily cut off on a lapidary's wheel, and another substituted in its place.

This accident, however, may be foreseen, and avoided by supporting the work in the same manner as do the workers in porcelain, who execute work infinitely more dnlicate than the porcelain work of dentists.

"§ 3.—*Artificial Gums applied and forming a Base.*—If the teeth have been arranged and soldered with fine gold to a strap of platina, they may be imbedded in a sufficient quantity of the paste indicated in the preceding paragraph, so as to form a base, the thickness, length and size that is required to make an artistical piece of work. Thus we shall have a perfect imitation of the species of work done by M. de Chemant with the kind of mineral teeth which, at the present time, are acknowledged as the best imitation of nature.

"I very frequently use this mode of procedure for the manufacturing of pieces destined for the inferior maxillary. When it is necessary to use ligatures, I make holes in the piece. And when I intend soldering to it metallic pieces, I insert cramps of attachment (platinas) in the proper places.

"§ 4 —*Artificial Gums inserted between a Metallic Base, or Plate, and the Teeth.*—After having mounted a set of teeth on a plate, I apply the above mentioned paste to the backs of the teeth and between their interstices, so as only to form the points of the gums in front, and an inclinec plane in the back part of the teeth. When there exists a great absorption of the alveolus, I first solder pivots to the stamped plate; after all the teeth have been ground to their proper dimensions, they are arranged on the pivots by means of the little eyes (platinas) inserted in their backs, taking care that the masticating surface shall correspond with the antagonising teeth, after which they are to be soldered. They are then to be tried in the mouth; there correct their defects, which can readily be done, for it is only necessary to bend the pivots, in order to give the teeth the required inclination. I may add, by the way, that this mode is of very great advantage. Afterwards solder to them a band or circular bar, which, whilst it retains them in their proper places, adds surprisingly to the solidity of the whole work.

"Now fill in the space between the base of the teeth and the plate with the paste. This must be baked before the enamel is put on, for as much as flaws always open in many places in baking. These cracks are to be filled with new paste. The armature (plate) may be extremely thin,and consequently will be much lighter in pieces of considerable extent, and requiring little height; particularly when there shall be some sound teeth remaining, inasmuch as they should always be preserved for the benefit of the patient.

"The paste which is placed in the interstices unites with the surrounding parts, forming one solid mass of the whole, imparting much beauty to the work.

"To recapitulate, it will be seen that by the addition of a mineral flux, the porcelain material (paste) is rendered much more fusible than that of our porcelain manufactories. Dentists may therefore avail themselves of this information, as well for the purpose of baking in furnances of their own as for imitating the gums. In the last case, the teeth, which have been baked in the furnace of the porcelain manufacturer, will undergo no alteration when exposed to the heat which it is requisite to employ in their own furnaces.

"Lastly, with the porcelain paste softened and prepared in the manner I have spoken of, to which has been added the proper oxides, sets of teeth can be made in the same method as done by M. de Chemant; and they have the advantage of being transparent. I employ this method particularly when I wish to make pieces containing only molar teeth. This preparation stands the heat of the soldering lamp perfectly well, and is no more liable to crack than the (calliodontes) ordinary porcelain teeth themselves.

§5.—*Application of the Gum Color.*—I have already shown the manner of painting the gums upon the enamel according to M. de Chemant's method; but experience has taught me that the friction of the lips and the secretions of the mouth dims at first, then gradually destroys the superficial coloring.

"I had recourse to the following method: I baked the piece in biscuit, that is to say without putting on the enamel, then I painted on the color and vitrified it. In withdrawing it from the fire it was dull, but I gave it brilliancy by covering the painting with a glaze made of the crystal of *Mont Cenis*, which I caused to fuse evenly. The saliva having no longer any direct action on the color, I should have been satisfied with this method, if it had not required several bakings and much time; I therefore adopted the following method:

"In the first place I bake the base; if any crevices are opened in it I fill them with paste a little more fusible than the first. and now I give it a coat of enamel, the fusibility of which corresponds with the degree of heat required to semi-vitrify the base. I incorporate in it a small quantity of the muriate of gold (ter-chloride of gold). I place it in the muffle, and stop off the heat as soon as I have obtained the required shade, for it is important to bear in mind that the longer the work is submitted to the action of the heat, the lighter will be the color."

